# Feasibility of Using Biochar as an Eco-Friendly Microfiller in Polymer Concretes

**DOI:** 10.3390/polym14214701

**Published:** 2022-11-03

**Authors:** Maja Kępniak, Kamil Załęgowski, Piotr Woyciechowski, Jędrzej Pawłowski, Jakub Nurczyński

**Affiliations:** Department of Building Materials Engineering, Faculty of Civil Engineering, Warsaw University of Technology, 00-637 Warsaw, Poland

**Keywords:** biochar, polymer concrete, eco-friendly microfiller

## Abstract

The circular economy includes, among other things, the use of waste materials. One such material is biochar, which should not be used as a fuel because its combustion generates large amounts of air pollution. This study evaluates the feasibility of using biochar as a partial filler in a polymer concrete. The components of the polymer concretes used in this study were vinyl-ester resin, traditional microfiller—quartz powder, waste microfiller—biochar and quartz aggregate with grain sizes up to 2 mm. The quartz aggregate was dosed at a constant rate of 1458 kg/m^3^ of concrete, whereas the dosage of resin and microfiller was formulated according to the experimental plan for mixtures and executed based on the volume of the remaining space: resin (65–85%), quartz powder (5–35%) and biochar (0–10%). The effects on the setting process, the consistency of the fresh composite mix and the flexural and compressive strengths were investigated. The study revealed significant deterioration of technological parameters (over 15% of biochar content makes a mixture unworkable) and slight deterioration of mechanical ones (flexural strength did not change significantly, and the compressive strength decreased by up to 15%). These results indicate that, despite some limitations, the use of biochar as an alternative pro-ecological filler is possible.

## 1. Introduction

Increasing climate challenges and a limited supply of new natural resources for construction projects have shifted research toward sustainability. The concept of the circular economy is one of the effective ways to achieve a long-term, sustainable construction sector [1,2,3,4]. Circular economy includes, among other things, the use of waste materials that have not yet been used [5,6,7]. One such material is biochar, which should not be used as a fuel because its combustion generates large amounts of air pollution. Polymer concrete is widely used in the building industry. Its wide application results from its performance properties including high strength, excellent corrosion resistance, frost resistance, good abrasion behavior, rapid hardening and easy preparation [8,9,10,11,12,13]. Another consideration is its low carbon footprint compared to cement concrete. There is usually no reactivity between the surrounding polymer matrix and aggregate particles [14]. Therefore, the replacement of natural aggregates in the production of polymer concrete is a very effective method of waste material disposal, which preserves natural resources. Powder waste materials are often used as an essential microfiller in polymer concretes [15,16,17,18,19,20,21,22,23]. Even irregularly shaped materials with large specific surface areas that would be difficult to use in cement concrete can be utilized in polymer concretes [24,25]. 

The search for greener alternatives for concrete production has drawn attention to the use of biochar. It is a solid type of waste obtained as a by-product from the thermochemical conversion of biomass to bioenergy under controlled conditions [26]. More precisely, according to the guidelines of the European Biochar Certificate [27], biochar is defined as charcoal resulting from biomass pyrolysis, a process in which organic substances are decomposed at temperatures between 350 °C and 1000 °C in a low oxygen environment. The resulting material is typically characterized by high specific surface areas, high porosity and good absorption capacity [28].

Due to the ability of biochar to bind a large fraction of carbon, as well as its porous structure and water retention properties, it is currently mainly used in agriculture, as a soil additive. Its properties and effectiveness as a material for carbon sequestration also make it potentially interesting for use as a component of concretes. Research examining the use of biochar as an additive in cementitious materials has been conducted and have shown very promising outcomes [29,30,31,32,33,34,35]. Biochar has also been considered as an alternative, eco-friendly microfiber reinforcement in polymer composites [36,37] because it is more beneficial than natural fibers. The properties of biochar can be altered by modifying its fabrication conditions [38] to achieve greater compatibility with the polymer matrix [37,39]. In general, carbon fillers are introduced into polymers to improve mechanical, thermal, electrical and chemical corrosion resistance properties compared to metal-filled composites [37,40]. The thermal stability of the obtained biochar composites is higher than that of natural fiber composites [39]. Improving these properties is desirable for many applications [32]. The resulting properties of the composites are determined by many factors, such as matrix and filler characteristics, matrix–filler interactions and dispersion of filler particles in the polymer matrix [41]. 

Studies conducted on mixtures with finer grain sizes, classified by the authors as nanocomposites, indicate a beneficial effect of the use of biochar as an effective reinforcement [42,43].

In this publication, the basic properties of polymer composites containing biochar as a partial volumetric substitution of mix components, including workability and strength, were investigated. The authors also focused on the color aspects of the designed composites, in relation to their use as a repair material meeting aesthetic requirements. 

The purpose of dosing waste biochar in polymer concrete is to replace natural raw materials in the form of quartz powder, the preparation of which generates a high carbon footprint and consumes natural resources. In turn, the use of biochar will enable the disposal of waste. The aim of this study is to create a composite with a reduced impact on the environment, yet without deteriorating the properties of the composite. 

The novelty of the presented work is in the potential applications of untreated biochar as a partial microfiller in polymer concrete; additionally, another new aspect is the possibility of applying volumetric experimental plan to ternary mixtures to assess the possibility of using biochar in polymer concrete.

## 2. Materials and Methods

The polymer used to prepare all composites in this study was synthetic vinyl-ester resin (Aropol M 105 TB; Ineos Composites, Miszewo, Poland, European Union) of low viscosity (350 ± 50 mPa·s at 25 °C), and high flexural strength and tensile strength (declared by the producer as, respectively, 110 MPa and 75 MPa). Therefore, concretes made from this resin should retain high mechanical strength in long-term exploitation, even when exposed to aggressive environments. The chemical formula of vinyl-ester (the polyester modified by introducing the fragments of the corresponding bisphenol epoxy resin to the structure of the molecule) could be found in [44].

**Figure 1:**
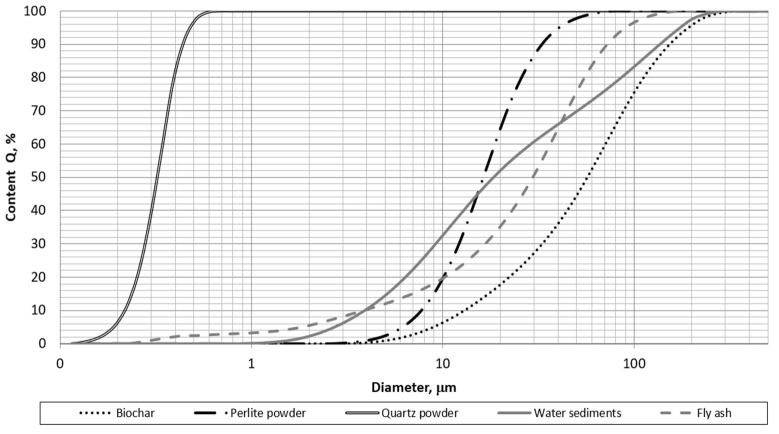
Grain size distribution of biochar—dotted line in comparison with other materials used as polymer concrete microfillers.

**Figure 2:**
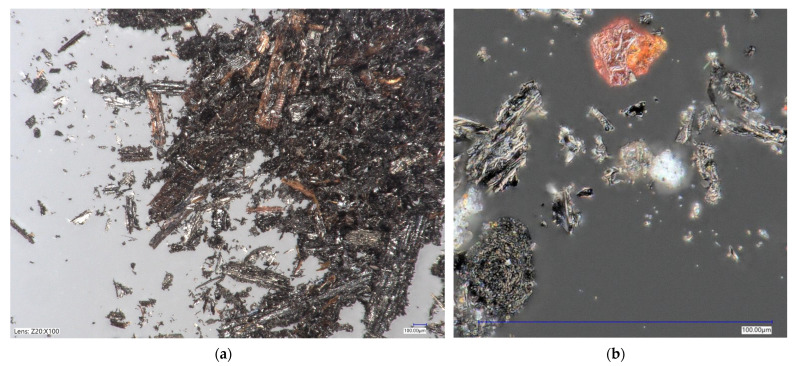
Optical microscope view of biochar: (**a**) overall picture; (**b**) shape of the single grain.

Traditionally used quartz powder (SKSM, Sobótka, Poland) and waste powder in the form of biochar (Fluid S.A., Sędziszów, Poland) were used as a microfiller. Testing with a laser particle size analyzer (Horiba, Irvine, CA, USA) showed that about 85% of the particles were below 120 µm in diameter (Table 1, Figure 1). This result does not exclude the use of biochar as a filler for polymer composites. The shape of the grains was also quite promising in terms of low specific surface area. Although most grains were not spherical in shape, most of them had smooth surfaces without much branching that could increase resin demand (Figure 2). CEN standard sand EN 196-1 (Kwarcmix, Tomaszów Mazowiecki, Poland) was used to produce the mortar for the strength tests.

The grain size of biochar differed significantly from that of a traditional polymer composite filler, such as quartz powder. However, a comparison of the granulometric characteristics of biochar with other successfully used waste-derived fillers showed that, in general, while its particles were larger, 85% of the particles were smaller than 120 µm, indicating that the majority of biocarbon can be considered a microfiller (Table 1, Figure 1 and Figure 2).

To assess the initial behavior of biochar in polymer composites, the following tests were carried out: the course of setting, the effect on consistency, the flexural and compressive strengths. 

**Figure 3:**
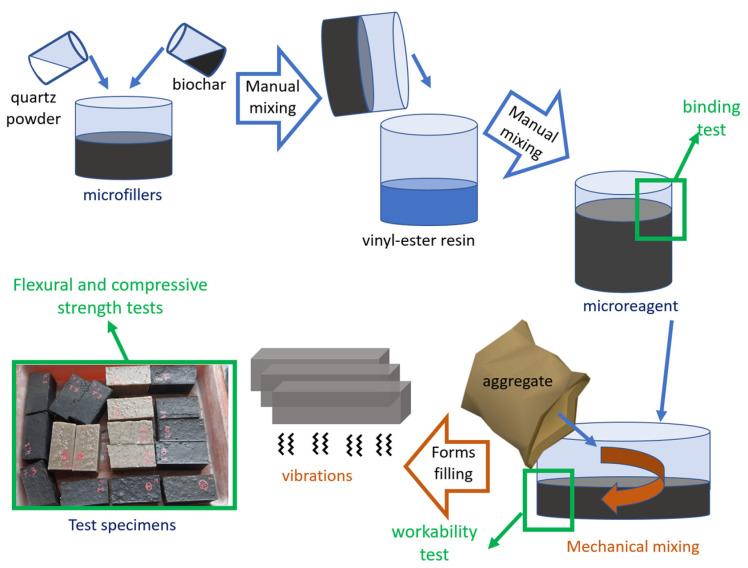
Manufacturing process.

Sample preparation took place in a three-stage process. In the first step, the microfiller mixture was prepared, followed by the addition of microfiller mixture to the resin, and then final mechanical mixing with quartz sand. The mixture was then placed in two layers in the molds and vibrated for 5 s until the mold was tightly filled (Figure 3).

The course of binding was analyzed on microreagent samples (resin and microfiller mixture). The temperature of the microreagent was measured at an interval of 1 min. The workability time, gelation time, hardening time, curing time and maximum temperature were evaluated based on the experiment and sample observations according to the guidelines described in [45].

One of the basic technological parameters is the consistency of the mix. In this study, the mortar flow diameter was measured according to the PN-85/B-04500 standard. The measurement was based on forming a truncated cone from the mortar and then subjecting it to 15 shocks. Two perpendicular spread diameters were measured and then their average was calculated.

Flexural and compressive strength tests were carried out on beams measuring 40 mm by 40 mm by 160 mm, prepared according to EN 196-1. 

**Figure 4:**
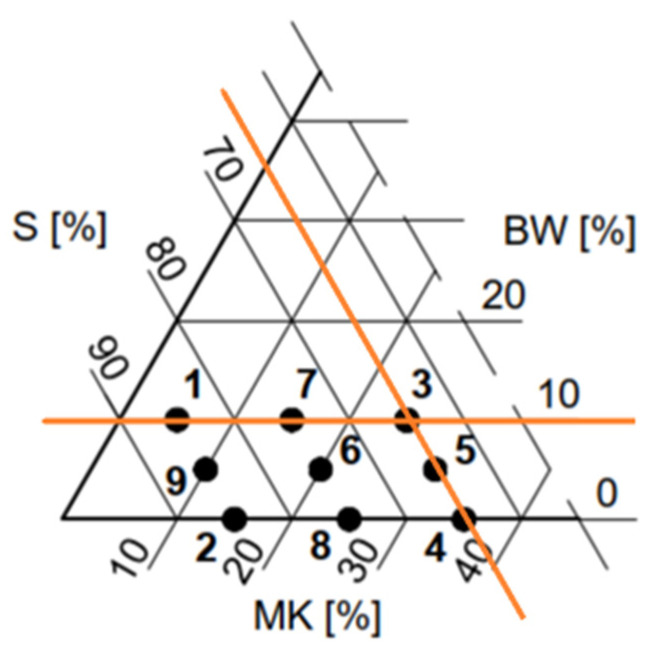
Experimental plan with limitations (orange lines): S—resin, BW—biochar, MK—quartz powder.

In order to determine the effect of biochar on polymer composites, an experimental plan was prepared, based on the standards for ternary mixtures. The dosage of sand was applied as a constant. The experimental plan was intended for the component proportions in the micro slurry. Therefore, the amount of resin (S), biochar (BW), and quartz powder (MK) were applied as input variables. In preliminary studies, the maximum and minimum dosages of the individual components were determined, so that the mixture could be workable and samples could be formed. Consequently, the experimental plan had limitations (Figure 4, Table 2). In the experimental plan, the volumetric variation in the dosage of components was assumed.

## 3. Results

As part of the study to assess the suitability of biochar in polymer composites, the following technological characteristics were investigated: the workability time; gelation time; hardening time; curing time; maximum temperature; consistency and mechanical characteristics—compressive and flexural strength. 

### 3.1. The Course of Binding

**Figure 5:**
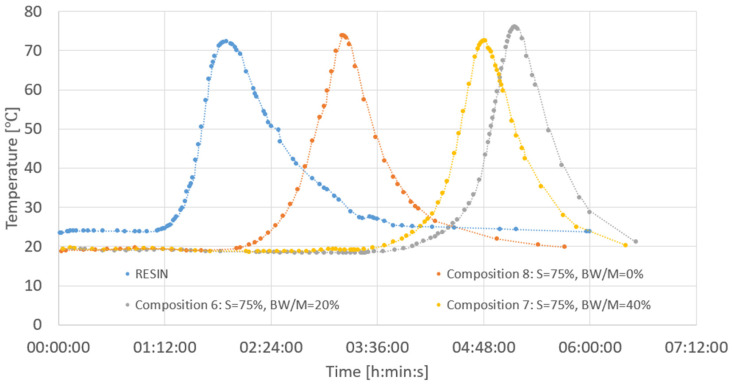
Binding course of the analyzed composites.

Testing the course of setting began shortly after mixing the ingredients, and it was performed on a 200 mL sample. The shortest curing time was observed for the resin itself (less than 2 h). The enrichment of the mixture with microfiller resulted in a longer setting process. The composite consisting of 75% resin and 25% quartz powder (without biochar) had the shortest workability time, gelation time and hardening time (Figure 5). The setting process started 1 h later than in the case of pure resin composite. The curing time curves for the two compositions in which the quartz powder was partially replaced by biocarbon were the longest—the curing time was about 5 h in both cases. The highest temperature (and the longest curing time at the same time) was observed for the composition no. 6, in which the BW/M ratio was 0.2 (Table 3).

### 3.2. Consistency

**Figure 6:**
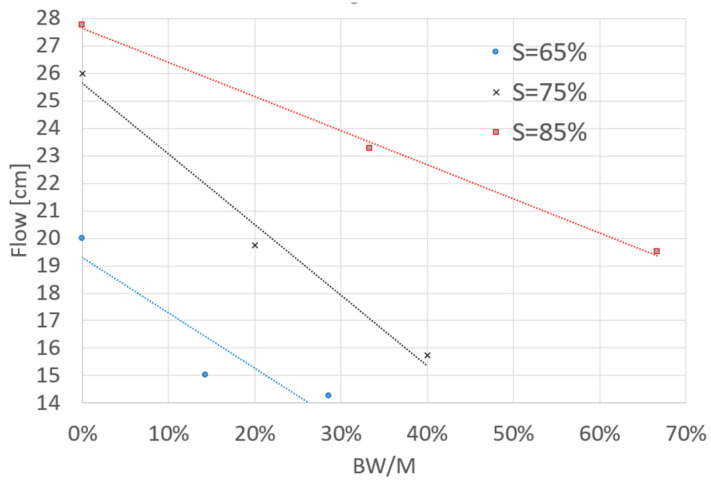
Flow of a fresh composite as a measure of workability as a function of the level of biochar content and the level of polymer content.

The consistency of the mixture was assessed according to the procedure for measuring the plasticity of construction mortars (according to the PN-EN 1015-3 standard), just after mixing the ingredients. A truncated cone (bottom diameter 100 mm, top diameter 70 mm, height 60 mm) was formed on the flow table. The fresh composite thus formed was subjected to 15 generative shakes by lifting and dropping the measuring table to a height of 10 mm at a rate of 1 per second. The diameter of the resulting flow was then measured. The results are summarized in Table 4. In this study, it was noted that with an increase in biochar content in the mix, there was a decrease in the measured flow diameter. It can also be seen that the greater the reduction in the mix flowability, the smaller the resin impaction in the fresh composite. Each of the resulting mixtures created within the experimental plan could be considered as sufficiently workable. They tightly filled the test molds and showed no segregation (Figure 6).

### 3.3. Flexural Strength

To test the flexural strength of the composite, three rectangular specimens, measuring 40 mm by 40 mm by 160 mm, were made for each composition included in the experimental plan. The specimens were tested according to the EN 196-1 standard. The three-point loading method was used. The distance between the supports was 100 mm. The specimen was placed in the apparatus with one side face on the supporting rollers and with its longitudinal axis normal to the supports. The load was applied vertically by means of the loading roller to the opposite side face of the prism and increased smoothly at the rate of (50 ± 10) N/s until fracture. The flexural strength was calculated. The results are summarized in Table 4.

**Figure 7:**
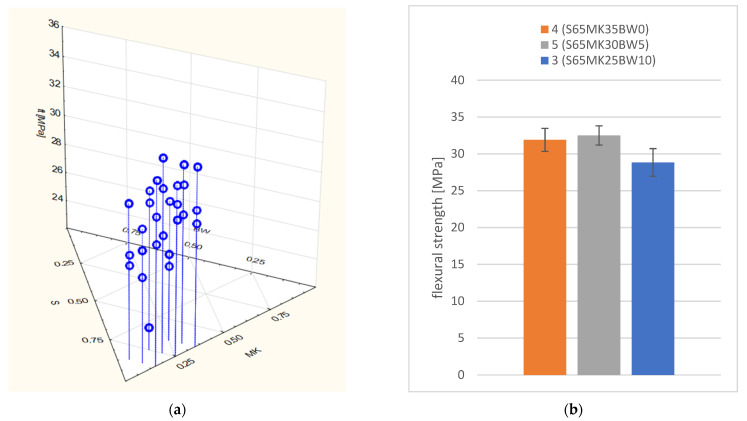
Flexural strength of the tested composites: (**a**) dependence in the experimental plan; (**b**) dependence on biochar content.

For the obtained flexural strength results the search for the regression equation describing the dependence between flexural strength and adopted variables was unjustified. The differences between the results were within the standard deviations of the measurements made for one composition. Therefore, it can be concluded that modifications to the composition of the composite within the established limits of the experimental plan do not have a statistically significant effect on the result of the flexural strength assessment (Figure 7).

A dosage of biochar up to 5% (S65MK30BW5) does not result in a significant reduction in flexural strength. A 10% biochar dosage (S65MK25BW10) is likely to have lower flexural strengths than non-biochar modified composites due to poorer workability and thus more difficult compaction of the fresh composite (Figure 7b). However, despite the lower average flexural strength value, it should be noted that the differences between the compositions with different levels of biochar dosage: S65MK35BW0, S65MK30BW5 and S65MK25BW10 are within the standard deviations of the results for one composition.

### 3.4. Compressive Strength

To test the compressive strength of the composite, six specimens were made for each composition in the experimental plan. The specimens were tested according to the EN 196-1 standard. The compressive strength test was carried out on prism halves that had been broken earlier in the flexural strength test. The compression surface was determined by steel plates measuring 40 mm by 40 mm. The prism halves were centered laterally to the plates of the machine within ± 0.5 mm, and longitudinally so that the end face of the prism overhung the plates by about 10 mm. The load was increased smoothly at the rate of 2000 N/s over the entire load application until fracture. Next, the compressive strength was calculated and results are summarized in Table 4.

**Figure 8:**
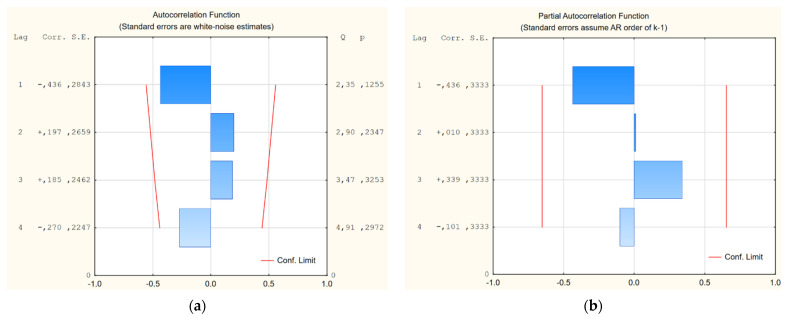
Residual number of the equation of compressive strength: (**a**) autocorrelation function; (**b**) partial autocorrelation function.

The regression equation describing the dependence of strength on composition was obtained from the compressive strength results (Equation (1)). The coefficient of determination (R^2^ = 0.80 and MAPE = 1.58%) was calculated. The partial autocorrelation function and autocorrelation function of the residual number of the equation of compressive strength were analyzed (Figure 8):

(1) $$f_{c}=94.51\cdot S+115.61\cdot MK+21.13\cdot BW$$

A summary of the results of the compression strength analysis, together with the forecast values is summarized in Table 5. In most compositions, the forecast can be considered as good.

**Figure 9:**
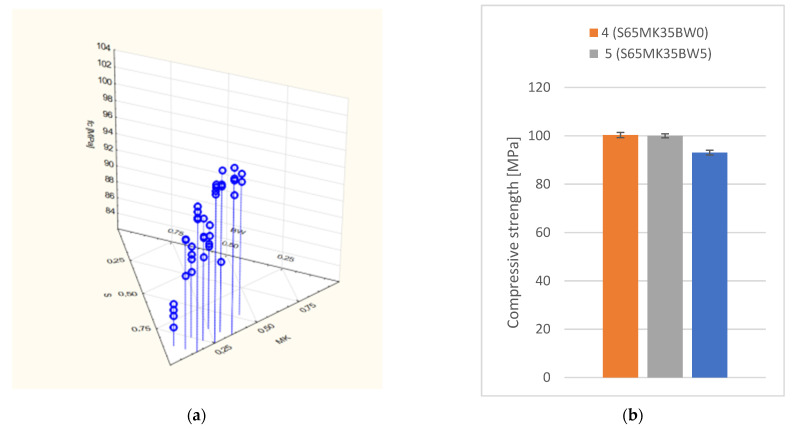
Compressive strength of the tested composites: (**a**) dependence in the experimental plan; (**b**) dependence on biochar content.

The resulting model indicates that the introduction of biochar into the polymer matrix yields a slight reduction in compressive strength. This effect is less pronounced when biochar replaces part of the polymer (a decrease of 5–7%) than when it replaces part of the quartz filler (a decrease of 10–15%). A dosage of biochar up to 5% (S65MK30BW5) does not result in a significant reduction in compressive strength. A 10% biochar dosage (S65MK25BW10) is likely to have lower compressive strength than non-biochar modified composites, due to poorer workability and thus more difficult compaction of the fresh composite (Figure 9b).

## 4. Discussion and Conclusions

This study examined the potential use of biochar as a partial microfiller for concrete-like polymer composites. Such a solution would reduce the consumption of mineral raw materials, which would be both environmentally and economically beneficial. The grain size characteristics of biochar show mostly positive features in the context of concrete polymers. More than 85% of biochar by mass are grains smaller than 0.125 mm. A disadvantage may be unburned biomass fragments, the amount of which depends on the parameters of the pyrolysis process carried out. 

The substitution of quartz powder with biochar, even in a small amount, increases the setting time of the resin mixture. This is because the biochar particles are larger than the grains of the dust fraction of the powder, which results in a longer setting time of the polymer matrix near the microfiller grains. 

The biochar does not impair the flexural strength of the polymer composite. The results obtained after flexural tests range from 27 to 35 MPa, which is in line with the expected range for polyester resin-based composites. The concretes of all tested compositions have a high compressive strength. An increase in the proportion of biochar in the composite resulted in a slight deterioration of this mechanical characteristic. What is important in the context of compressive strength is the total microfiller content, which should be around 35% of the proportion in the composite. The small substitution of quartz powder by biochar then results in a negligibly small decrease in compressive strength, compared to the large-scale economic benefits. The research conducted here supports the conclusion that biochar can be used as a partial substitute for quartz powder in the role of microfiller for polymer composite. Statistical analysis showed that replacing the traditional microfiller in a volume amount of about 15%, with a total share of quartz powder and biocarbon of 35% in the composite, produced very positive results with acceptable consistency and workability.

Due to the variability of the waste, it is necessary to repeat the tests for material obtained under different manufacturing conditions. Further research is also required to determine the durability of the composite under conditions of chemical aggression. 

## Figures and Tables

**Table 1:** Selected characteristics of waste and non-waste polymer composite fillers.

Microfiller	Specific Surface Area, cm^2^/cm^3^	Average Diameter, µm	Mode Diameter, µm	Mean Diameter, µm	Grains under 120 µm, %
Quartz powder	7847	10.19	10.83	10.19	100
Perlite powder	4392	18.83	18.49	18.83	100
Water sediments	6046	46.25	10.81	43.25	86
Fly ash	9718	35.16	41.92	35.16	98
Biochar	1921	71.93	72.29	67.22	85

**Table 2:** Compositions of analyzed mortars according to the experimental plan.

Composition Symbol	Volume Proportions, %			Mass Proportion, kg/m^3^			
	Resin (S)	Quartz Powder (MK)	Biochar (BW)	Resin	Quartz Powder	Biochar	Sand
1 (S85MK5BW10)	85	5	10	451	60	79	1458
2 (S85MK15BW0)	85	15	0	451	180	0	1458
3 (S65MK25BW10)	65	25	10	345	299	79	1458
4 (S65MK35BW0)	65	35	0	345	419	0	1458
5 (S65MK30BW5)	65	30	5	345	359	39	1458
6 (S75MK20BW5)	75	20	5	398	239	39	1458
7 (S75MK15BW10)	75	15	10	398	180	79	1458
8 (S75MK25BW0)	75	25	0	398	299	0	1458
9 (S75MK10BW5)	75	10	5	451	120	39	1458

**Table 3:** Binding characteristics of the composite depending on the proportion of biochar.

Hardening Process	Resin	Composition 8 (S = 75%; BW/M = 0%)	Composition 6 (S = 75%; BW/M = 20%)	Composition 7 (S = 75%; BW/M = 40%)
Workability time, min	70	120	210	210
Gelation time, min	95	160	260	250
Hardening time, min	120	195	310	290
Curing time, min	240	330	400	400
Maximum temperature, °C	72.3	73.7	72.5	75.5

**Table 4:** The result of consistency, flexural and compressive strength.

Composition Number	Volume Proportions, %			Test Results		
	Resin (S)	Quartz Powder (MK)	Biochar (BW)	Flow, cm	Flexural Strength, MPa	Compressive Strength, MPa
1 (S85MK5BW10)	85	5	10	19.5	30.0 ± 1.8	85.9 ± 0.3
2 (S85MK15BW0)	85	15	0	27.8	32.2 ± 1.7	98.4 ± 1.0
3 (S65MK25BW10)	65	25	10	14.3	28.8 ± 1.9	93.0 ± 1.0
4 (S65MK35BW0)	65	35	0	20.0	31.9 ± 1.6	100.3 ± 1.1
5 (S65MK30BW5)	65	30	5	15.0	32.5 ± 1.3	100.0 ± 0.9
6 (S75MK20BW5)	75	20	5	19.7	32.7 ± 2.1	94.4 ± 1.6
7 (S75MK15BW10)	75	15	10	15.7	32.3 ± 0.4	91.7 ± 1.1
8 (S75MK25BW0)	75	25	0	26.0	32.3 ± 1.0	100.2 ± 0.5
9 (S75MK10BW5)	75	10	5	23.5	29.5 ± 1.3	94.0 ± 1.8

**Table 5:** Result of the compressive strength—measured and predicted.

Composition Number	Volume Proportions, %			Statistical Verification		
	Resin (S)	Quartz Powder (MK)	Biochar (BW)	Mean Compressive Strength, MPa	Predicted Compressive Strength, MPa	Prediction Error, %
1 (S85MK5BW10)	85	5	10	85.9	88.2	2.7
2 (S85MK15BW0)	85	15	0	98.4	97.7	−0.7
3 (S65MK25BW10)	65	25	10	93.0	92.4	−0.6
4 (S65MK35BW0)	65	35	0	100.3	101.9	1.6
5 (S65MK30BW5)	65	30	5	100.0	97.2	−2.8
6 (S65MK20BW15)	75	20	5	94.4	85.6	−9.3
7 (S75MK15BW10)	75	15	10	91.7	90.3	−1.5
8 (S75MK25BW0)	75	25	0	100.2	99.8	−0.4
9 (S75MK10BW5)	75	10	5	94.0	83.5	−11.2

## Data Availability

The data presented in this study are available on request from the corresponding author.
